# Electrical Impedance Tomography-Guided Synergistic Intervention of Respiratory Muscle Training and Postural Adjustment for Lung Recruitment in Critically Ill Patients With Respiratory Failure

**DOI:** 10.33549/physiolres.935744

**Published:** 2026-08-01

**Authors:** Hongfei WANG, Zhu LIN, Hongmei GAO

**Affiliations:** 1 Department of Intensive Care Unit, Tianjin First Central Hospital, Tianjin, China

**Keywords:** Electrical impedance tomography, Lung recruitment, Respiratory muscle training, Postural adjustment, Respiratory failure

## Abstract

Lung collapse and ventilation heterogeneity are common in mechanically ventilated critically ill patients. Electrical impedance tomography (EIT) enables real-time bedside monitoring of regional ventilation and may facilitate individualized respiratory management. This study evaluated the effect of EIT-guided respiratory muscle training (RMT) combined with postural adjustment on lung recruitment in patients with respiratory failure. In this single-center, prospective, randomized controlled trial, 150 patients were randomly assigned to three groups: the EIT collaborative group (EIT-guided RMT+postural adjustment), the EIT posture group (EIT-guided positioning only), and the conventional collaborative group (RMT+postural adjustment without EIT). The primary endpoint was the change in end-expiratory lung impedance (EELI). Secondary outcomes included oxygenation, respiratory mechanics, ventilation homogeneity, and clinical outcomes. Data were analyzed using ANOVA and linear mixed-effects models. The interventions were applied for up to 5 consecutive days after randomization or until ICU discharge, whichever occurred first. After 5 days, EELI increased by 18.4±7.6 % in the EIT collaborative group, which was significantly greater than the increases observed in the EIT posture group (9.1±6.2 %) and the conventional collaborative group (3.7±5.8 %) (P<0.001). CL % decreased markedly without a significant increase in OD %, while CoV and GI index indicated improved ventilation uniformity. The EIT collaborative group also showed favorable trends toward improved oxygenation and respiratory mechanics, along with shorter ventilation duration and higher extubation success rates. A significant interaction between EIT supervision intensity and intervention level was confirmed (F=6.45, P=0.011). No serious adverse events occurred. These findings suggest that EIT-guided RMT combined with postural adjustment may enhance lung recruitment and ventilation homogeneity, with potential benefits for respiratory mechanics and selected short-term clinical outcomes. Real-time EIT feedback may provide a safe, individualized strategy for respiratory rehabilitation in critically ill patients.

## Introduction

Respiratory failure is a common and life-threatening condition among critically ill patients, frequently requiring mechanical ventilation to maintain adequate gas exchange [1,2]. However, prolonged mechanical ventilation is associated with several complications, including ventilator-induced lung injury (VILI), atelectasis, and diaphragmatic dysfunction [3,4]. These pathophysiological changes contribute to ventilation heterogeneity, impaired oxygenation, and delayed weaning, ultimately leading to poor clinical outcomes and prolonged hospitalization [5]. Consequently, optimizing lung recruitment while minimizing overdistension and regional ventilation imbalance remains a major challenge in respiratory management of critically ill patients [6].

Respiratory muscle training (RMT) has been proposed as an adjunctive intervention to enhance respiratory muscle strength, facilitate ventilator weaning, and support spontaneous breathing [7]. Meanwhile, postural adjustments such as prone or lateral positioning are recognized for improving ventilation distribution and oxygenation in patients with acute respiratory distress syndrome (ARDS) and other forms of respiratory failure [8,9]. Despite their individual benefits, the combined application of these two interventions in clinical practice has largely relied on empirical experience and lacks objective, real-time guidance to optimize intervention strategies and reduce regional lung stress.

Electrical impedance tomography (EIT) is a noninvasive, radiation-free bedside imaging modality capable of continuously visualizing regional lung ventilation and aeration distribution in real time [10]. By quantifying parameters such as end-expiratory lung impedance (EELI), collapse ratio (CL %), and overdistension ratio (OD %), EIT enables dynamic assessment of lung recruitment, derecruitment, and ventilation homogeneity during mechanical ventilation [11,12]. Recent studies have demonstrated that EIT can guide individualized ventilator adjustments and prone positioning, thereby improving patient safety and clinical outcomes [13,14]. However, whether EIT-guided management can be extended to synergistically optimize both postural adjustments and active respiratory muscle engagement has not yet been systematically investigated.

Based on these considerations, we hypothesized that integrating RMT and postural management under real-time EIT guidance could produce a synergistic effect on lung recruitment in critically ill patients with respiratory failure. This multimodal strategy aims to dynamically redistribute regional ventilation, enhance diaphragmatic activation, and prevent localized collapse or overdistension. Accordingly, we conducted a prospective, randomized controlled study to evaluate the efficacy and safety of EIT-guided combined RMT and postural adjustment compared with either intervention alone or conventional management. By integrating EIT monitoring into a synergistic intervention framework, this study sought to determine whether real-time feedback could improve ventilation homogeneity and respiratory mechanics, enhance oxygenation, and potentially translate into improved clinical outcomes.

## Methods

### Study design and participants

This single-center, prospective, randomized controlled clinical study was conducted in the Department of Intensive Care Medicine at our hospital between June 2019 and June 2025. The study protocol was approved by the ethics committee of Tianjin First Central Hospital (Approval No. 2023DZX11-1), and written informed consent was obtained from all subjects. The study was performed in accordance with the principles of the Declaration of Helsinki. A total of 150 critically ill patients receiving invasive mechanical ventilation were enrolled and randomly assigned to three groups using a computer-generated randomization sequence created by an independent statistician: the EIT collaborative group (EIT-guided RMT combined with postural changes, n=50), the EIT posture group (EIT-guided postural management only, n=50), and the conventional collaborative group (RMT combined with postural adjustment without EIT guidance, n=50). Block randomization with a block size of six was used to ensure balanced group allocation. Allocation concealment was maintained using sequentially numbered, sealed, opaque envelopes prepared by a researcher who was not involved in patient recruitment or outcome assessment. All study interventions and protocol-specified assessments were conducted for up to 5 consecutive days after randomization or until ICU discharge, whichever came first.

### Sample size estimation

The sample size was calculated based on the expected difference in the rate of change in end-expiratory lung impedance (EELI) from a preliminary pilot study. Assuming a mean difference of 10 % between the EIT collaborative group and the conventional collaborative group, with a standard deviation of 15 %, a two-sided significance level of α=0.05, and a statistical power of 80 % (1-β=0.80), the required sample size was calculated to be 45 patients per group. Considering a potential dropout or loss to follow-up rate of 10 %, the final sample size was increased to 50 patients per group, resulting in a total sample size of 150 participants.

### Inclusion and exclusion criteria

The inclusion criteria were as follows: (1) Aged between 18 and 85 years; (2) Diagnosis of acute or chronic respiratory failure requiring invasive mechanical ventilation for ≥48 hours; (3) Suitable for EIT monitoring and RMT; (4) Clinically stable and suitable for intervention (defined as FiO_2_≤0.6, PEEP≤12 cmH_2_O); (5) An expected duration of mechanical ventilation ≥72 hours to ensure feasibility of the intervention.

The exclusion criteria included: (1) Severe neuromuscular disease or respiratory muscle injury; (2) Presence of chest drainage or extensive chest wall trauma; (3) Pregnancy or severe obesity (BMI>35 kg/m^2^); (4) Inability to tolerate prone position or RMT; (5) Other clinical conditions considered by the treating physicians to be unsuitable for study participation.

### Intervention methods

Patients in the EIT collaborative group received a combined intervention consisting of RMT and postural adjustment under EIT guidance. The EIT posture group received EIT-guided postural management only, while the conventional collaborative group underwent combined RMT and postural adjustment without EIT guidance.

EIT monitoring was performed using a PulmoVista 500 electrical impedance tomography system (Dräger Medical GmbH, Lübeck, Germany). The system provided continuous real-time visualization of regional ventilation distribution and automatically calculated key parameters, including EELI, CL %, and OD %. Protocol-specified EIT monitoring was performed during the study intervention period (up to 5 consecutive days after randomization or until ICU discharge). If patients remained mechanically ventilated beyond day 5, any further EIT monitoring was performed according to routine clinical practice and was not included in the study analysis.

RMT was performed using HLO-GJ13A external diaphragmatic pacing device (Arahelio Biotechnology Corp., China). After skin preparation, the smaller electrode was placed over the distal one-third of the lateral border of the sternocleidomastoid muscle, while the larger electrode was positioned at the second intercostal space along the midclavicular line. Diaphragmatic pacing was applied at a rate of 9 cycles per minute with a stimulation frequency of 40 Hz for 30–40 minutes per session, one to three sessions per day. Stimulation intensity was set to the maximal level tolerated by the patient. All RMT sessions were supervised by professional therapists. Training adherence and performance were recorded, and the RMT load index was calculated as: daily load intensity × effective repetitions × number of intervention days.

Postural management strategies were individualized according to lung ventilation distribution assessed by EIT. Both the EIT collaborative and EIT posture groups underwent alternating prone, semi-prone, or lateral positions. Each prone session lasted at least 4 hours, with a total daily prone duration of ≥8 hours. Postural adjustment was performed according to a predefined protocol based on EIT-derived indices. When EIT parameters indicated a CL % >20 % or OD % >10 %, clinicians were prompted to modify the patient’s position to redistribute regional ventilation and promote lung recruitment. After repositioning, EIT monitoring was repeated to reassess ventilation distribution and confirm improvement. All adjustments were implemented by trained ICU clinicians according to the standardized intervention protocol.

### Primary and secondary outcomes

The primary outcome was the change in lung recruitment, assessed by the variation in EELI relative to baseline, together with changes in the CL % and OD %. EELI and related parameters were automatically calculated by the EIT system to quantify regional lung aeration and recruitment efficiency.

Secondary outcomes included oxygenation parameters, assessed by the arterial oxygen partial pressure to inspired oxygen fraction ratio (PaO_2_/FiO_2_), respiratory mechanics (static compliance [Crs] and driving pressure [ΔP]), and ventilation homogeneity indices (center of ventilation [CoV] and global inhomogeneity [GI] index). In addition, major clinical outcomes were recorded, including the duration of invasive mechanical ventilation, extubation success rate, ICU length of stay, and 28-day mortality.

### Process and safety evaluation

Process indicators included the duration of tolerated prone positioning, the completion rate and compliance with RMT, and the number of intervention adjustments triggered by EIT feedback. Safety assessments included the occurrence of hypoxemic episodes, hemodynamic instability, skin pressure injuries, and accidental catheter dislodgement. All adverse events occurring during the study were recorded and reviewed by the research team to evaluate the safety and tolerability of the interventions.

### Data collection and quality control

All EIT measurements were performed by the same trained investigator, and RMT sessions were supervised by a designated therapist to ensure protocol consistency. Daily data were independently entered into electronic spreadsheets by two research assistants and cross-checked to verify accuracy and completeness. Outcome assessors responsible for collecting and validating clinical outcome data were blinded to group allocation to minimize detection bias.

### Statistical analysis

Statistical analyses were performed using SPSS 26.0 and Python 3.11. Continuous variables were tested for normality using the Shapiro-Wilk test and are expressed as mean±standard deviation (χ̄±s). Differences among the three groups were assessed using one-way analysis of variance (ANOVA), followed by Bonferroni post hoc tests. Categorical variables were expressed as frequencies and percentages, and were compared using the χ^2^ test or Fisher’s exact test when the expected frequency was <5. Repeated measures data with time effects were analyzed using linear mixed-effects models, with group, time, and their interaction included as fixed effects. To explore the potential synergistic effect between the intensity of EIT supervision and the level of intervention implementation, multivariable linear regression and linear mixed-effects models were constructed. These models incorporated the EIT supervision index, intervention execution level, and their interaction term, while adjusting for potential confounders including age, sex, BMI, APACHE II score, ARDS status, baseline PEEP, and baseline EIT parameters. All continuous variables were standardized prior to modeling after normality assessment, and model parameters were estimated using robust standard errors to calculate 95 % confidence intervals (CI). For statistical analysis, study groups were coded and the statistician remained blinded to group allocation until the analysis was completed. A P-value < 0.05 was considered statistically significant.

## Results

### Baseline characteristics

Baseline demographic and clinical characteristics were comparable among the three groups. There were no statistically significant differences in sex, age, BMI, smoking history, primary diagnosis, APACHE II score, mode of mechanical ventilation, FiO_2_, PEEP, or baseline EIT parameters, including EELI, CoV, GI index, CL %, and OD % (P>0.05) (Supplementary Table S1).

### Change in EELI relative to baseline (lung recruitment index)

EELI values increased from baseline in all three groups, with the greatest improvement observed in the EIT collaborative group. According to the study protocol, physiological outcomes were evaluated from baseline to day 5 or until ICU discharge, whichever occurred first. By day 5, the mean increase in EELI relative to baseline was 18.42±7.56 % in the EIT collaborative group, 9.12±6.21 % in the EIT posture group, and 3.74±5.83 % in the conventional collaborative group, with a significant difference among groups (P<0.001) (Table 1).

Linear mixed-effects modeling revealed a significant interaction between time and group (F=12.53, P<0.001). The collaborative intervention was identified as an independent predictor of EELI improvement (β=0.15, 95 % CI: 0.08–0.22, P<0.001), indicating that EIT-guided RMT combined with postural adjustment was more effective in promoting lung recruitment (Table 2).

### Changes in CL % and OD %

After 5 days of intervention, the CL % in the EIT collaborative group decreased significantly compared with baseline (−6.30±2.10 %, P<0.001). A smaller reduction was observed in the EIT posture group (−3.10±2.50 %, P<0.05), whereas no significant change was detected in the conventional collaborative group (P>0.05). The OD % increased slightly in all groups compared with baseline. However, these changes were not statistically significant (P>0.05). Intergroup comparison revealed that the EIT collaborative group showed the greatest reduction in CL % (F = 22.46, P < 0.001), suggesting that EIT-guided RMT combined with postural adjustment achieved more effective recruitment of collapsed lung regions without inducing additional overdistension (Table 3).

### Changes in CoV and GI Index

Following the intervention, both CoV and GI index improved to varying degrees in all three groups, with the most pronounced changes observed in the EIT collaborative group (Table 4). In this group, CoV increased from 46.18±5.47 % at baseline to 49.98±5.32 % after intervention (P<0.001), while the GI index decreased from 0.42±0.08 to 0.36±0.07 (P<0.001). The EIT posture group also showed significant improvement (P<0.05), whereas no significant changes were observed in the conventional collaborative group (P>0.05). Intergroup comparison showed that the EIT collaborative group exhibited the greatest improvement in both indices (F=5.36 and F=7.90, P<0.001), suggesting that the combined EIT-guided intervention substantially enhanced ventilation homogeneity.

### Changes in oxygenation and respiratory mechanics

After 5 days of intervention, the EIT collaborative group showed significant improvements in oxygenation and respiratory mechanics (Table 5). The PaO_2_/FiO_2_ ratio increased from 168.5±45.6 to 243.7±52.1 mmHg (P<0.001), which was significantly higher than that in both the EIT posture group and conventional collaborative group (P<0.001). ΔEtCO_2_ decreased from 6.7±2.5 to 2.9±1.8 mmHg (P<0.001), indicating improved ventilation-perfusion matching. In addition, Crs increased from 34.8±6.5 to 41.3±6.8 ml/cmH_2_O (P<0.001), while ΔP decreased from 16.2±2.9 to 13.1±2.7 cmH_2_O (P<0.05). Overall, these results indicate that the EIT-guided combination of RMT and postural adjustment effectively improved oxygenation and respiratory mechanics.

### Clinical outcomes

Patients in the EIT collaborative group generally showed more favorable short-term clinical outcomes across groups, although not all endpoints reached statistical significance (Supplementary Table S2). The duration of invasive mechanical ventilation was 7.3±3.5 days in the EIT collaborative group, which was significantly shorter than in the EIT posture group (9.1±4.2 days) and the conventional collaborative group (10.0±4.7 days, P=0.012). The extubation success rate was also highest in the EIT collaborative group (78.0 %), compared with 62.0 % in the EIT posture group and 54.0 % in the conventional collaborative group (P=0.038). Similarly, ICU length of stay was shorter in the EIT collaborative group (12.6±4.3 days) than in the EIT posture group (13.9±5.0 days) and the conventional collaborative group (15.1±5.2 days, P=0.041).

Although the 28-day mortality was lower in the EIT collaborative group (10.0 %) compared with the EIT posture group (14.0 %) and the conventional collaborative group (20.0 %), the difference did not reach statistical significance (P=0.363).

### Process indicators

During the intervention period, patients in the EIT collaborative group demonstrated greater tolerance for prone positioning, while overall compliance remained comparable across groups. The mean tolerated duration of prone positioning was 13.40±3.10 h/day in the EIT collaborative group, which was significantly longer than that in the EIT posture group (10.80±2.90 h/day) and the conventional collaborative group (7.20±2.60 h/day), with a significant overall difference among groups (F=32.15, P<0.001). Pairwise comparison further showed a significant difference between the EIT collaborative and conventional collaborative groups (t=10.16, P<0.001). The positional compliance rates were 93.20 %, 91.60 %, and 88.40 % in the EIT collaborative, EIT posture, and conventional collaborative groups, respectively, with no significant difference among groups (χ^2^=1.46, P=0.23). The completion rate of RMT, assessed only in the EIT collaborative and conventional collaborative groups, was 93.20 % and 91.60 %, respectively, with no significant difference (χ^2^=0.18, P=0.67). A total of 126 EIT-triggered feedback adjustment events were recorded during the study period. Of these, 76 events (60.3 %) occurred in the EIT collaborative group and 50 events in the EIT posture group, showing a significant difference between groups (χ^2^=6.35, P=0.01). These findings indicate that real-time EIT monitoring provided meaningful guidance for individualized postural adjustment and optimization of RMT intensity.

### Safety

Overall, the interventions were well tolerated during the study period. The incidence of hypoxemic events was 4.0 %, 6.0 %, and 8.0 % in the EIT collaborative, EIT posture, and conventional collaborative groups, respectively, with no significant differences among groups (χ^2^=0.99, P=0.61). No severe hemodynamic instability occurred during the intervention period. No clinically significant RMT-related adverse events, such as arrhythmias or paradoxical breathing, were observed. A small number of patients reported transient mild discomfort during RMT sessions. However, these symptoms were self-limited and did not require discontinuation of training. Minor events included two cases of mild skin erythema and one case of accidental catheter dislodgement in the collaborative groups, all of which resolved after appropriate management without serious complications. No additional EIT belt-related complications were observed beyond the reported mild transient skin erythema. These findings indicate that EIT-guided RMT combined with postural adjustment was safe and feasible in critically ill patients, without introducing additional risk.

### Synergistic effect analysis

**Fig. 1:**
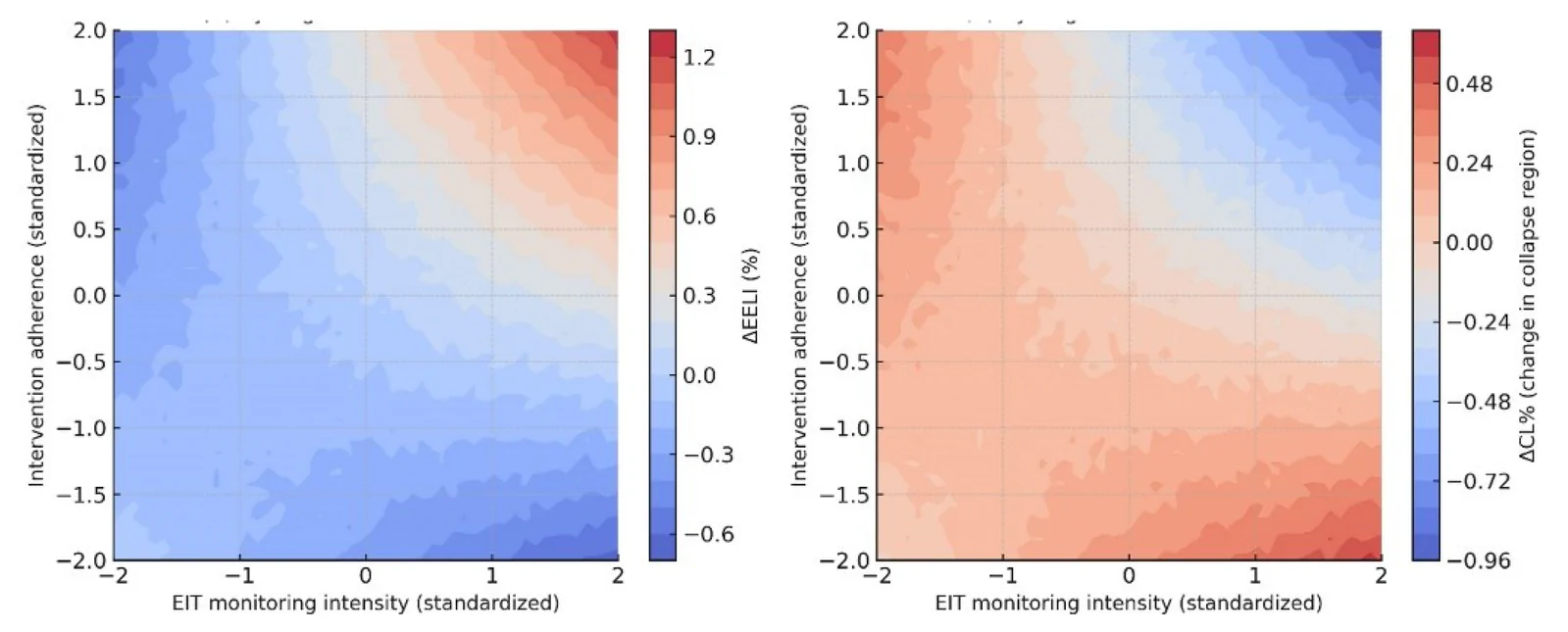
Interaction effects of EIT supervision intensity and intervention performance level on lung-recruitment indicators. (A) ΔEELI: A positive interaction was observed between EIT supervision intensity and intervention performance (β=0.15, P<0.001), indicating that higher intervention performance under greater EIT supervision was associated with more pronounced improvement in EELI. (B) ΔCL (%): A negative interaction was identified (β=–0.12, P=0.002); the combination of high EIT supervision and high intervention performance markedly reduced the proportion of collapsed regions, suggesting that their synergistic effect enhances lung recruitment without increasing overdistension risk. Color gradients represent model-predicted effect magnitudes: red areas indicate greater positive improvement (higher EELI or lower CL %), whereas blue areas indicate smaller or attenuated effects.

To further investigate the contribution of EIT supervision to lung recruitment during the combined intervention, an EIT supervision intensity index and a collaborative intervention performance index (a standardized composite score of RMT load and prone positioning duration) were constructed using intervention records from the EIT collaborative and EIT posture groups. These indices, along with their interaction term, were incorporated into multivariate linear regression and linear mixed-effects models, adjusting for covariates including age, sex, BMI, APACHE II score, ARDS status, baseline PEEP, and baseline EIT parameters (Supplementary Table S3). The analysis revealed a significant positive interaction between EIT supervision intensity and intervention performance in predicting EELI improvement (β=0.15, 95 % CI: 0.08–0.23, t=3.87, P<0.001) (Fig. 1A), suggesting that more frequent EIT monitoring strengthened the synergistic effect of RMT and postural adjustment on lung recruitment. Similarly, analysis of ΔCL (%) also demonstrated a significant negative interaction (β=−0.12, 95 % CI: −0.20 to −0.05, t=−3.14, P=0.002) (Fig. 1B), indicating that enhanced EIT supervision facilitated the reduction of collapsed lung regions without increasing overdistension. The mixed-effects model validation further confirmed that the interaction terms time × EIT supervision and intensity × intervention level remained statistically significant (F=6.45, P=0.011).

Overall, these findings suggest that the contribution of real-time EIT feedback extends beyond simple quantitative monitoring. By enabling individualized adjustment of intervention parameters, EIT may facilitate more precise lung recruitment and improve the overall efficacy of respiratory rehabilitation. Consequently, EIT-guided respiratory muscle activation combined with postural modulation may represent an important mechanism for improving ventilation homogeneity and optimizing lung recruitment.

## Discussion

This study demonstrated that the combination of RMT and postural adjustment under real-time EIT guidance significantly enhanced lung recruitment, improved ventilation homogeneity, and optimized respiratory mechanics in mechanically ventilated critically ill patients. Compared with conventional management, EIT-guided collaborative intervention resulted in a greater increase in EELI, a more pronounced reduction in CL %, and improved oxygenation indices, indicating clear physiological benefits. Although the EIT collaborative group also exhibited favorable trends in clinical outcomes, including shorter ventilation duration, higher extubation success rates, and reduced ICU length of stay, these findings should be interpreted with caution. Notably, the difference in mortality did not reach statistical significance, and the present study was not powered to detect differences in mortality outcomes. Therefore, the observed improvements in extubation success and survival should be considered hypothesis-generating rather than definitive evidence of benefit on major clinical endpoints. These findings suggest that dynamic EIT supervision enables individualized optimization of both active and passive lung recruitment strategies, offering a feasible and potentially effective approach for respiratory rehabilitation in critically ill patients.

Previous studies have highlighted the value of EIT in monitoring regional ventilation and guiding ventilator settings or prone positioning in patients with hypoxemic respiratory failure [15]. However, most existing protocols primarily rely on passive recruitment maneuvers and often overlook the contribution of respiratory muscle activity to lung aeration. Previous evidence suggests that maintaining or training respiratory muscle function can promote more homogeneous ventilation and improve diaphragmatic efficiency during mechanical ventilation [16,17]. In the present study, integrating EIT monitoring with RMT and postural modulation not only enhanced lung recruitment but also reduced the risk of overdistension, as reflected by the concurrent improvement in the GI index and CoV. Moreover, the observed reduction in CL % and stability of OD % suggests that EIT-guided adjustments facilitated effective reopening of collapsed regions without inducing excessive inflation. These findings are consistent with prior reports supporting the clinical relevance of these EIT-derived indices in assessing regional ventilation distribution [18,19]. Importantly, our results further expand current knowledge by demonstrating that EIT may function not only as a diagnostic imaging modality but also as a dynamic monitoring and feedback tool for guiding interactive respiratory interventions.

Real-time EIT feedback has been shown to facilitate individualized titration of ventilator settings and postural strategies by detecting regional overdistension and collapse [14,20]. Building on this concept, the synergistic interaction between EIT supervision intensity and intervention performance observed in our study provides mechanistic insight into how individualized feedback enables dynamic adjustment of RMT load and postural configuration in response to evolving lung mechanics. Continuous visualization of regional aeration allows clinicians to fine-tune intensity, optimize positioning strategies, and identify the point at which further recruitment yields diminishing physiological benefit [15,21,22]. Such a precision-guided approach promotes adequate regional expansion while minimizing ventilator-associated mechanical stress. Moreover, the observed improvement in Crs and reduction in ΔP may reflect improved lung elastance and mechanical efficiency, potentially resulting from reduced regional collapse and more homogeneous ventilation distribution.

From a clinical perspective, the EIT-guided combined strategy appeared both effective and safe. The intervention was associated with greater tolerance for prone positioning while overall compliance remained comparable across groups. These findings support the feasibility of integrating EIT into routine ICU practice as a noninvasive, real-time decision-support tool to enhance the safety and effectiveness of multimodal respiratory interventions. Although improvements were observed in several short-term clinical outcomes, including ventilation duration and extubation success, these results should be interpreted in the context of the study design and sample size. Larger, adequately powered multicenter trials are required to determine whether these physiological benefits translate into improvements in major clinical outcomes such as extubation success, ICU length of stay, or survival.

Nevertheless, several limitations should also be acknowledged. First, this was a single-center study with a relatively limited sample size, which may restrict the generalizability of the findings. Second, due to the nature of the interventions, blinding of bedside clinicians and therapists was not feasible, which may have introduced performance bias. However, outcome assessors responsible for collecting clinical outcome data, as well as the statistician performing the analysis, were blinded to group allocation, thereby reducing the risk of detection bias. Third, despite the use of standardized protocols, variations in RMT intensity and tolerance to postural interventions among patients may have introduced additional variability. Fourth, the study was powered primarily to detect physiological changes in lung recruitment rather than differences in hard clinical outcomes such as extubation success or 28-day mortality. Accordingly, the observed differences in these clinical outcomes, particularly survival, should be interpreted as exploratory and hypothesis-generating. Finally, this study focused primarily on short-term physiological and clinical outcomes and long-term respiratory muscle function and post-extubation recovery were not evaluated. Future multicenter studies with extended follow-up and mechanistic analyses, such as diaphragm ultrasound or electromyography, are warranted to further elucidate the mechanisms through which EIT-guided interventions improve lung mechanics and clinical prognosis.

## Conclusion

In summary, this study provides novel evidence that real-time EIT guidance enhances the synergistic effects of RMT and postural adjustment on lung recruitment in critically ill patients with respiratory failure. By enabling individualized optimization of both ventilatory mechanics and respiratory muscle activation, the EIT-guided combined strategy may represent a promising approach for precision respiratory rehabilitation. Nevertheless, the potential impact of this intervention on major clinical outcomes requires confirmation in larger, adequately powered multicenter studies.

## Supplementary Information



## Figures and Tables

**Table 1:** Changes in EELI at different time points among the three groups (χ̄±s, Ω·cm)

Time point	EIT collaborative group (n=50)	EIT posture group (n=50)	Conventional collaborative group (n=50)	F (between groups)	P (between groups)
*Baseline*	1352.47±210.36	1338.52±216.91	1346.83±205.27	0.05	0.94
*Day 1*	1437.02±215.41*	1386.49±210.18*	1369.34±204.12	1.40	0.24
*Day 3*	1520.73±226.25**	1429.95±218.36*	1390.38±206.54	4.73	<0.05
*Day 5 or before ICU discharge*	1601.35±232.14**	1459.34±219.42*	1397.12±207.61	11.32	<0.001

* P < 0.05,

** P < 0.01 vs baseline within the same group.

**Table 2:** Results of linear mixed-effects model analysis (predictors of EELI improvement)

Variable	β	95% CI	t	P
*Group (Collaborative vs Conventional)*	0.15	0.08~0.22	4.72	<0.001
*Group (Posture vs Conventional)*	0.07	0.02~0.12	2.63	0.010
*Time (Day 5 vs Baseline)*	0.12	0.06~0.19	4.15	<0.001
*Group × Time interaction*	0.09	0.04~0.14	3.98	<0.001
*Age*	−0.01	−0.03~0.01	−1.03	0.31
*APACHE II score*	−0.02	−0.04~0.00	−2.01	0.046
*ARDS (Yes vs No)*	0.05	−0.01~0.11	1.68	0.095

**Table 3:** Comparison of changes in CL% and OD% among the three groups (χ̄±s)

	Parameter	EIT collaborative group (n=50)	EIT posture group (n=50)	Conventional collaborative group (n=50)	F (between groups)	P (between groups)
*CL%*	Baseline (collapsed regions)	15.62±3.74	15.28±3.91	15.94±3.67	0.38	0.68
	Day 5 or before ICU discharge	9.32±3.21**	12.18±3.64*	15.14±3.59	34.85	<0.001
*OD%*	Baseline (overdistended regions)	5.84±2.13	5.72±2.08	5.96±2.25	0.15	0.85
	Day 5 or before ICU discharge	7.04±2.24	6.72±2.18	6.76±2.32	0.30	0.74

* P < 0.05,

** P < 0.01 vs baseline within the same group.

**Table 4:** Comparison of changes in CoV and GI index among the three groups (χ̄±s)

	Parameter	EIT collaborative group (n=50)	EIT posture group (n=50)	Conventional collaborative group (n=50)	F (between groups)	P (between groups)
*CoV (%, dorsal direction)*	Baseline	46.18±5.47	45.82±5.23	45.96±5.62	0.05	0.94
	Day 5 or before ICU discharge	49.98±5.32**	47.72±5.12*	46.56±5.48	5.36	<0.05
*GI index*	Baseline	0.42±0.08	0.43±0.09	0.43±0.08	0.23	0.78
	Day 5 or before ICU discharge	0.36±0.07**	0.40±0.08*	0.42±0.08	7.90	<0.001

* P < 0.05,

** P < 0.01 vs baseline within the same group.

**Table 5:** Comparison of oxygenation and respiratory mechanics before and after intervention among the three groups (χ̄±s)

Parameter	Time point	EIT collaborative group (n=50)	EIT posture group (n=50)	Conventional collaborative group (n=50)	F (between groups)	P (between groups)
*PaO* *_2_* */FiO* *_2_* * (mmHg)*	Baseline	168.51±45.62	172.33±44.92	170.45±46.17	0.08	0.91
	Day 5 or before ICU discharge	243.74±52.12**	205.82±47.65*	182.68±50.27	18.97	<0.001
*ΔEtCO* *_2_* * (mmHg)*	Baseline	6.76±2.58	6.83±2.44	6.63±2.62	0.07	0.92
	Day 5 or before ICU discharge	2.90±1.83**	4.92±2.01*	5.89±2.25	28.03	<0.001
*Crs (ml/cmH* *_2_* *O)*	Baseline	34.84±6.55	35.11±6.34	34.91±6.77	0.02	0.97
	Day 5 or before ICU discharge	41.38±6.81**	38.26±6.41*	35.83±6.54	8.91	<0.001
*ΔP (cmH* *_2_* *O)*	Baseline	16.29±2.92	15.97±3.08	16.16±2.82	0.14	0.86
	Day 5 or before ICU discharge	13.17±2.76*	14.42±2.80	15.47±2.93	8.27	<0.001

* P < 0.05,

** P < 0.01 vs baseline within the same group.
