# Supplementary material for: Electrical Impedance Tomography-Guided Synergistic Intervention of Respiratory Muscle Training and Postural Adjustment for Lung Recruitment in Critically Ill Patients With Respiratory Failure
Source: Physiol Res. 2026 Aug 1;75(4):759–69. doi: 10.33549/physiolres.935744 (PMC13580672; doi:10.33549/physiolres.935744)
Supplement: Supplementary file 1 [file PR75_759_Supplementary.pdf]

TABLE S1

Variable	EIT collaborative group (n=50)	EIT posture group (n=50)	Conventional collaborative group (n=50)	F/χ^2	P
Sex (male) [n,%]	33 (66.00)	32 (64.00)	34 (68.00)	0.17	0.91
Age (years)	58.24±14.53	59.78±13.87	57.92±15.31	0.23	0.79
BMI（kg/m^2）	23.42±3.57	23.68±3.44	23.12±3.29	0.33	0.71
Smoking history（n,%）	21 (42.00)	20 (40.00)	23 (46.00)	0.38	0.82
APACHE II score	18.60±4.52	19.12±4.64	18.24±4.39	0.47	0.62
ARDS cases [n,%]	28 (56.00)	26 (52.00)	27 (54.00)	0.16	0.92
Mechanical ventilation mode (invasive) [n,%]	35 (70.00)	34 (68.00)	36 (72.00)	0.19	0.90
FiO_2 (%)	46.80±10.25	47.52±9.87	45.96±9.61	0.31	0.73
PEEP (cmH_2O)	8.42±1.61	8.58±1.73	8.34±1.57	0.27	0.75
EELI (Ω·cm)	1352.47±210.36	1338.52±216.91	1346.83±205.27	0.05	0.94
CoV (%)	46.18±5.47	45.82±5.23	45.96±5.62	0.05	0.94
GI index	0.42±0.08	0.43±0.09	0.43±0.08	0.23	0.78
CL%	15.62±3.74	15.28±3.91	15.94±3.67	0.38	0.68
OD%	5.84±2.13	5.72±2.08	5.96±2.25	0.15	0.85

TABLE S2 Comparison of clinical outcomes among the three groups [mean±SD or n (%)]

Parameter	EIT collaborative group (n=50)	EIT posture group (n=50)	Conventional collaborative group (n=50)	F/χ^2	P
Duration of invasive ventilation (days)	7.37±3.51	9.18±4.25	10.05±4.76	5.28	<0.012
Successful extubation rate (%)	78.0	62.0	54.0	4.95	0.038
ICU length of stay (days)	12.65±4.33	13.92±5.07	15.14±5.22	3.24	0.041
28-day mortality (%)	10.0	14.0	20.0	2.02	0.363

TABLE S3 Baseline-adjusted regression analysis: interaction between EIT supervision intensity and intervention performance (standardized β)

Outcome variable	Predictor	β	95%CI	t	P
ΔEELI (%)	EIT supervision intensity	0.13	0.06~0.20	3.12	0.002
	Intervention performance level	0.17	0.09~0.25	4.38	<0.001
	Interaction (EIT supervision × intervention level)	0.15	0.08~0.23	3.87	<0.001
ΔCL (%)	EIT supervision intensity	-0.10	-0.17~-0.03	-2.56	0.012
	Intervention performance level	-0.14	-0.22~-0.07	-3.51	<0.001
	Interaction (EIT supervision × intervention level)	-0.12	-0.20~-0.05	-3.14	0.002
